# The crystal structure of the TolB box of colicin A in complex with TolB reveals important differences in the recruitment of the common TolB translocation portal used by group A colicins

**DOI:** 10.1111/j.1365-2958.2009.06808.x

**Published:** 2009-08-07

**Authors:** Ying Zhang, Chan Li, Mireille N Vankemmelbeke, Philip Bardelang, Max Paoli, Christopher N Penfold, Richard James

**Affiliations:** 1 Institute of Infection, Immunity and Inflammation, School of Molecular Medical Sciences, University of Nottingham, University ParkNottingham NG7 2RD, UK; 2 School of Pharmacy, Centre for Biomolecular Sciences, University of Nottingham, University ParkNottingham NG7 2RD, UK

## Abstract

Interaction of the TolB box of Group A colicins with the TolB protein in the periplasm of *Escherichia coli* cells promotes transport of the cytotoxic domain of the colicin across the cell envelope. The crystal structure of a complex between a 107-residue peptide (TA_1–107_) of the translocation domain of colicin A (ColA) and TolB identified the TolB box as a 12-residue peptide that folded into a distorted hairpin within a central canyon of the β-propeller domain of TolB. Comparison of this structure with that of the colicin E9 (ColE9) TolB box–TolB complex, together with site-directed mutagenesis of the ColA TolB box residues, revealed important differences in the interaction of the two TolB boxes with an overlapping binding site on TolB. Substitution of the TolB box residues of ColA with those of ColE9 conferred the ability to competitively recruit TolB from Pal but reduced the biological activity of the mutant ColA. This datum explains (i) the difference in binding affinities of ColA and ColE9 with TolB, and (ii) the inability of ColA, unlike ColE9, to competitively recruit TolB from Pal, allowing an understanding of how these two colicins interact in a different way with a common translocation portal in *E. coli* cells.

## Introduction

Many proteins of eubacterial or of eucaryotic origin contain stretches of > 30 amino acids that are natively (intrinsically) disordered regions (NDRs) (Ward *et al*., 2004). Native disorder occurs in regions with a high glycine content and a prevalence of charged or polar residues that preclude the formation of a hydrophobic core or stable three-dimensional fold. Such NDRs are important in many biological processes such as transcription, translation, intracellular signalling and host–pathogen interactions where their roles are to bind to other macromolecules to form complexes (Dyson and Wright, 2005). NDRs undergo disorder-order transitions on binding a partner molecule (Wright and Dyson, 1999). Coupling a folding transition to a protein–protein interaction may be functionally advantageous as it could contribute to the specificity of the molecular recognition, enhance the rate of the interaction, allow binding of one protein to several different target molecules, and could provide for large intermolecular interfaces within a relatively small protein (Gunasekaran *et al*., 2003). Recent studies with colicins have highlighted the important role of NDRs in the complex protein–protein interactions that drive cellular uptake of these proteins (Collins *et al*., 2002; Macdonald *et al*., 2004; Tozawa *et al*., 2005).

Colicins are plasmid-encoded, protein antibiotics that consist of three domains; an N-terminal translocation (T) domain, a central receptor-binding (R) domain and a C-terminal cytotoxic domain (James *et al*., 2002). The mechanism of cell killing by colicins is either by forming pores in the cytoplasmic membrane (e.g. colicins A, B, E1 or N) (Elkins *et al*., 1997); a non-specific DNase that belongs to the H-N-H family of homing endonucleases (colicin E2, E7, E8 and E9) and shows homology to DNases responsible for eucaryotic apoptosis (Walker *et al*., 2002); a 16S RNase (colicins E3, E4, E6 and cloacin DF13) (Boon, 1971; Senior and Holland, 1971); or an anticodon tRNase (colicin E5) (Ogawa *et al*., 1999). Colicin E2-E9 producing strains protect themselves against killing by producing a plasmid-encoded, immunity protein that forms a complex with its cognate cytotoxic domain on synthesis, for example ColE9/Im9 (Kleanthous *et al*., 1999). To facilitate import of their cytotoxic domains to their cellular site of action, group A colicins, such as the E colicins and colicin A (ColA), use the *tol*-dependent translocation system that consists of the TolQ, TolR, TolA, TolB and Pal proteins, and constitutes a transmembrane protein translocation portal or translocon (James *et al*., 1996; Zakharov *et al*., 2004).

The normal cellular function of the *tol* system in *Escherichia coli* is uncertain. It appears to play a role in maintaining the integrity of the cell envelope, transducing energy from the cytoplasmic membrane, and may form a dynamic subcomplex at constriction sites to promote the energy-dependent septal wall formation across invaginating peptidoglycan and inner membrane layers during cell division (Cascales *et al*., 2001; Goemaere *et al*., 2007; Gerding *et al*., 2007). TolB is a periplasmic protein, whose crystal structure was independently determined by two groups (Abergel *et al*., 1999; Carr *et al*., 2000), and is associated with the outer membrane via an interaction of its C-terminal β-propeller domain with the peptidoglycan-associated lipoprotein (Pal) (Bouveret *et al*., 1995), and with TolA via its N-terminal domain (Dubuisson *et al*., 2002). The TolB protein is essential for mouse-lethal infection by *Salmonella typhimurium* (Bowe *et al*., 1998). TolA is a 44 kDa periplasmic protein that is anchored in the cytoplasmic membrane via a single transmembrane region (TolAI) that is important for interactions with the TolQ and TolR proteins in the membrane (Germon *et al*., 2001). TolQ and TolR are transmembrane proteins that are involved in the pmf-dependent activation of TolA (Cascales *et al*., 2001) which shuttles energy from the inner to outer membrane through its association with Pal anchored to the outer membrane (Lloubès *et al*., 2001; Cascales *et al*., 2002). TolA spans the periplasm via its extended central domain (TolAII), and binds to both TolB (Dubuisson *et al*., 2002; Walburger *et al*., 2002) and Pal (Cascales *et al*., 2000) via its C-terminal domain (TolAIII). TolA has recently been shown to be important in the energy dependent loss of immunity protein from the ColE9/Im9 complex possibly through its interaction with TolB (Vankemmelbeke *et al*., 2009).

All the information required for translocation of colicins is found in their T domains. ColA interacts with TolB through a TolB box (DGTGW), that has been localized to residues 11–15 in the T domain of ColA (Bouveret *et al*., 1997) and shares high sequence homology with part of the TolB box of other group A colicins such as ColE9 (DGSGW) (Garinot-Schneider *et al*., 1997). The TolB box of ColE9 was later shown to be extended to include 15 contiguous residues with a tryptophan at residue 46 which is essential for anchoring the TolB box into the TolB canyon on the surface of the β-propeller domain (Hands *et al*., 2005; Loftus *et al*., 2006). The N-terminal translocation domain of ColE9 is natively disordered but contains clusters of interacting side-chains, one of which is centred around the TolB box residues 35–39 (DGSGW) (Collins *et al*., 2002; Macdonald *et al*., 2004; Tozawa *et al*., 2005). The Predictors of Natural Disordered Regions (PONDR®) programme (Romero *et al*., 1997) has predicted a high percentage of disorder over the first 150 residues of the T domain of ColA, particularly around the TolB box.

Both ColE9 and ColA bind to the BtuB outer membrane receptor and OmpF co-receptor and then recruit TolB, via their TolB boxes, during translocation, but they differ in their interactions with TolA. ColA recruits TolA during translocation (Bénédetti *et al*., 1991), whereas no physiological interaction has ever been demonstrated in yeast two hybrid, surface plasmon resonance (SPR) or isothermal titration calorimetry experiments between ColE9 and TolA. However, as an *E. coli tolA* mutant is resistant to killing by ColE9, the TolA protein must be required, possibly indirectly through its interaction with TolB, for the translocation of ColE9.

In this paper we have used site-directed mutagenesis data together with the elucidation of the cocrystal structure of a TolB box containing peptide consisting of the N-terminal 107 residues of ColA (TA_1–107_) bound to TolB to show that the TolB box of ColA consists of a 12-residue domain that binds to the β-propeller canyon of TolB. Comparison with the published cocrystal structure of a synthetic TolB box peptide of ColE9 bound to TolB (Loftus *et al*., 2006) indicates differences in the intermolecular binding patterns of both colicins to an overlapping binding pocket that helps to explain the observed differences in the affinity of binding and recruitment mechanisms of these two colicins for TolB. This datum is discussed in the context of a model for the translocation of these two colicins.

## Results

### Prediction of the TolB boxes of group A colicins

**Fig. 1:**
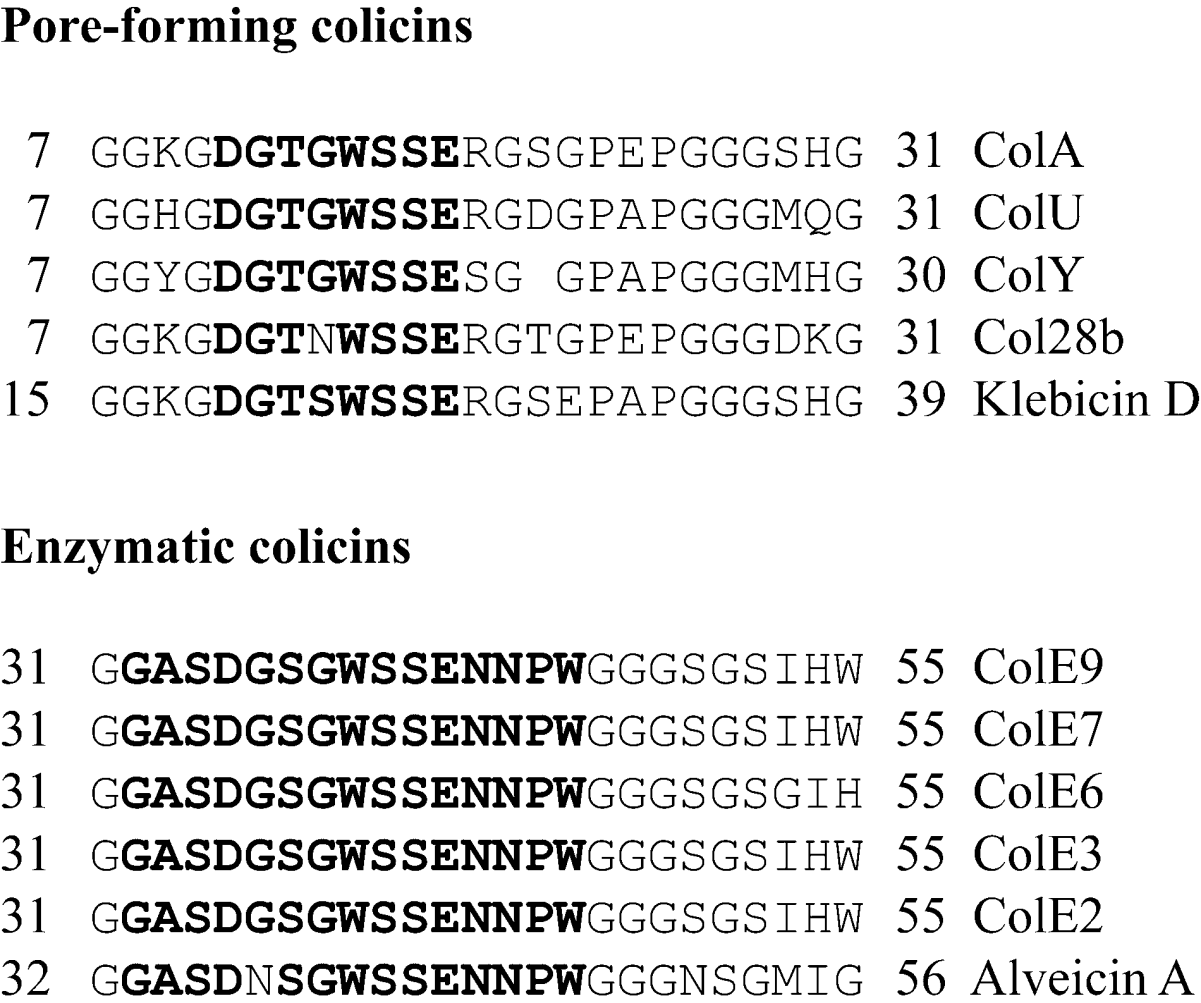
Alignment of residues of the TolB box region of pore-forming and enzymatic group A colicins. Residues of the extended TolB box of ColE9 and residues of the TolB box sequence that are conserved in the other colicin sequences are shown in bold. The residue numbers are indicated at the start and end of each sequence. A padding space has been introduced in the ColY sequence to optimize the alignment. Colicins A, U, Y and E2-E9 are produced by *E. coli*, Col28b is produced by *Serratia marcescens*, Klebicin D is from *Erwinia tasmaniensis* and Alveicin A is from *Hafnia alvei*.

The TolB box in ColE9 consists of 15 contiguous residues with the sequence 32-GASDGSGWSSENNPW-46 (Hands *et al*., 2005) (Fig. 1). Using deletion analysis, the TolB box of ColA was predicted to include residues 7–20 (Bouveret *et al*., 1998; Journet *et al*., 2001). Sequence alignment of the *tol*-dependent colicins showed that the DG(S/T)GWSSE residues are highly conserved in all of the enzymatic and pore-forming colicins (shown in bold in Fig. 1). Previous mutagenesis studies have shown that the G38 residue of ColE9 is not essential for activity (Garinot-Schneider *et al*., 1997), and that the S37 residue of ColE9 can be substituted by threonine without significant loss of function (Hands *et al*., 2005). We therefore assume that substitution of G14 of ColA to N14 or S14 of Col28b and Klebicin D respectively, and G36 of ColE9 to N34 of Alveicin A would similarly not affect biological activity. It is intriguing that ColA and the other pore-forming colicins are missing the last four residues of the extended TolB box of ColE9 (43-NNPW-46) which are replaced by RGSG in ColA. We have previously demonstrated that the N44A and W46A mutations resulted in loss of biological activity of ColE9, with the latter mutation abolishing the interaction with TolB (Hands *et al*., 2005).

### Mutagenesis of the TolB box of ColA

**Fig. 2:**
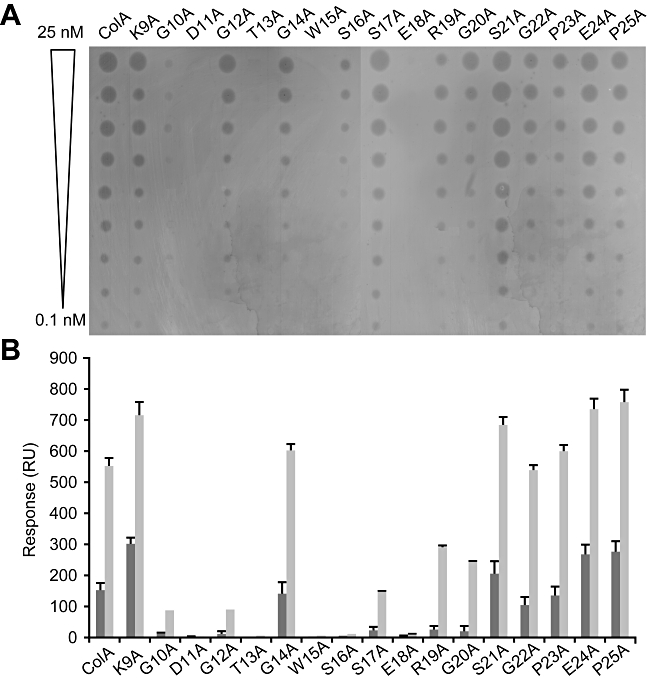
Alanine scanning mutagenesis of residues of the TolB box of ColA. A. Individual alanine mutations were engineered into ColA from Lys9 to Pro25 and their effect on the activity of ColA was determined using the spot test assay using doubling dilutions of purified proteins from 25 nM to 0.1 nM. B. The effect of each TolB box mutant of TA_1–107_ on the TA_1–107_–TolB interaction was determined by surface plasmon resonance and expressed in response units (RU) in the presence (grey bars) and absence (black bars) of 1 mM Ca^2+^.

Alanine scanning mutagenesis was conducted across the region from residue K9 to P25 to determine the extent of the TolB box of ColA and the contribution of individual residues to the binding of TolB. The biological activity of the alanine mutants in comparison with ColA (Fig. 2A) indicates that mutations in residues D11, T13, W15 or E18 abolished the biological activity of the mutant colicin. In contrast, alanine mutations of residues G12, G14, R19, G20 or G22 had little effect on biological activity while alanine mutations of residues S17 and S21 had no effect, and thus also presumably contribute little to the affinity of binding to TolB. We are, however, aware that the N44A mutation in the TolB box of ColE9 that abolished biological activity did not affect binding of the mutant colicin to TolB (Hands *et al*., 2005). Consequently, we determined the interaction of the ColA TolB box containing peptide (TA_1–107_) and all the TA_1–107_ alanine mutants, from K9 to P25 with TolB by SPR, in the presence or absence of Ca^2+^ (Fig. 2B), as Ca^2+^ has been shown previously to dramatically increase the binding affinity of the ColE9 TolB box for TolB (Loftus *et al*., 2006). Corrected sensorgrams and residual plots for the interaction of TolB with ColA and ColA E18A are shown in Fig. S1. In the presence of Ca^2+^ the affinity of binding (*K*_d_) of TA_1–107_ to TolB was 1.6 µM, compared with a value of 24.3 µM in the absence of Ca^2+^, indicating a similar enhancement by Ca^2+^ of the binding of ColA to TolB. Compared with TA_1–107_, alanine substitutions of residues K9 and S21 to P25 did not affect binding to TolB, whereas alanine substitutions of all residues between G10 and G20, with the exception of G14, resulted in either complete inhibition or significantly reduced binding to TolB when compared with that of ColA (Fig. 2). Interestingly, the ColA S16A mutant had partial biological activity using the spot test assay but a binding affinity to TolB that was very weak and similar to the inactive mutants, D11A, T13A, W15A and E18A. As the spot test assay only measures the activity of a colicin at the end of a 16 h incubation period, it is possible that this endpoint assay does not always fully differentiate between complexes that have different affinities or stabilities. Indeed, analysis of the biological activity of ColA containing the S16A mutation using a liquid growth assay over an 8 h period showed that the S16A mutation reduced the activity of ColA by, at least, 1000-fold compared with ColA, thus confirming the SPR data (Fig. S2). This datum indicates that the TolB box of ColA is located between residues G10 and G20 with many of the core residues being highly conserved between the *tol*-dependent enzymatic and pore-forming colicins (Fig. 1).

### Crystal structure of the TA_1–107_–TolB complex

ColE9 binds to TolB with an affinity of approximately 1 µM or 90 nM in the presence of Ca^2+^ (Hands *et al*., 2005; Loftus *et al*., 2006). Comparison with data on the interaction of TolB with the TolB box of ColA suggests an approximate 15–25-fold difference in affinities. In order to explain this difference, the structure of the complex of TA_1–107_ bound to TolB was determined so that a detailed comparison with the published structure of the TolB box of ColE9 in complex with TolB could be established. We rectify an error in previous reports of the amino acid sequence of TolB (PDB entry 1C5K) that include an additional incorrectly assigned methionine residue at the N-terminus.

**Fig. 3:**
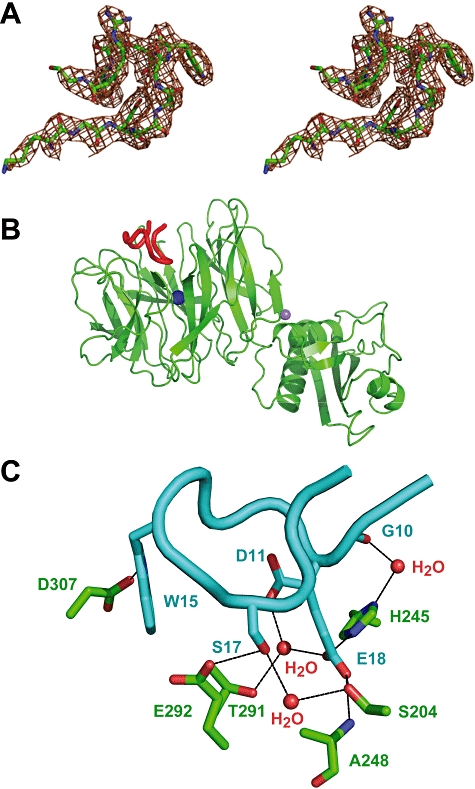
Structure of the TA_1–107_–TolB complex. A. Electron density map of residues 9–20 of TA_1–107_ contoured at 0.95 σ. Residues 9–20 were the only ColA residues with any electron density in the TA_1–107_–TolB cocrystal structure. Note, contouring was shown at 0.95 σ rather than 1 σ to provide a sharper representation of the electron density map given the resolution of the data. B. Structure of the TA_1–107_–TolB complex at 2.6 Å resolution showing the colicin binding site of the β-propeller domain. Also visible are one Ca^2+^ ion (blue) in the central channel of the β-propeller domain and one Na^+^ ion (violet) between the β-propeller and N-terminal α/β-domains. C. Intermolecular hydrogen bonding networks in the core region of the proximal half of the TA_1–107_–TolB peptide complex. ColA residues are shown in blue and the TolB residues are shown in green. The intermolecular hydrogen bonds are shown as black lines, three of which are mediated by water molecules.

The structure was refined to 2.6 Å resolution (see Table 1); and the model has excellent geometry with no Ramachandran outliers. The first electron density maps obtained with molecular replacement phases, using the known TolB structure (1C5K), clearly revealed strong additional peaks of density at the side of the β-propeller fold located furthest away from the smaller α/β N-terminal domain of TolB. Even though 107 amino acids of the translocation domain of ColA were included in the crystallization, only residues 9–20 could be resolved in the electron density maps (Fig. 3A). No additional peaks of density were observed at any stage of the model building and refinement, therefore the remaining residues of the TA_1–107_ polypeptide are assumed to be disordered in the solvent channels within the crystal lattice.

**Table 1:** X ray data collection and crystallographic refinement statistics for the TA_1–107_–TolB complex.

Data collection	
Wavelength (Å)	1.542
Space group	P1 2_1_ 1
Resolution range (Å)	30–2.6 (2.67–2.6)
Unit cell parameters	a = 61.96, b = 40.17, c = 80.87, α = γ 90°, β = 97.18°
No. of unique reflections	12220
Total no. of observations	44504
Redundancy	3.6 (3.7)
Mean *I/*σ(I)	13.3 (3.7)
*I/*σ(I)	7.7 (2.2)
Completeness (%)	97.9 (96.6)
Rmerge (%)	9.5 (34.7)
Refinement	
Resolution range (Å)	30–2.6
R_factor_ (%)	19.3
R_free_ (%)	25.7
rmsd bond lengths (Å)	0.015
rmsd angles (°)	1.77
Average B-factor (protein, Å^2^)	17
No. of protein atoms	3107
No. of water and ions atoms	70

The electron density was of sufficiently good quality to allow the placement of the colicin sequence with little ambiguity. However, in the absence of very high-resolution data, we also refined, as tests, a structural model with the ColA polypeptide fitted in the reverse order (Fig. S3). Difference maps and R_free_ statistics gave indisputable indications that this interpretation was incorrect. Our final colicin model fitted the density very well, with clean difference maps. TolB, as previously described (Abergel *et al*., 1999; Carr *et al*., 2000), is composed of an α/β domain at the N terminus and a six-bladed β-propeller domain at the C terminus with a tunnel through the centre of the β-propeller. TA_1–107_ binds within the larger of the cavities formed at the ends of the central tunnel of the TolB β-propeller domain (Fig. 3B), at the same location as the ColE9 TolB box peptide binds in the structure of the TE9_pep32–47_–TolB complex (PDB entry 2IVZ) (Loftus *et al*., 2006). Refinement of two small, independent peaks of positive density using water, monovalent and divalent cations suggested that one Ca^2+^ ion is present in the central channel of the β-propeller and a Na^+^ ion is present at the interface between the N-terminal α/β domain and β-propeller domain (Fig. 3B). The structure of TolB in the complex is very similar to that of the free TolB structure (1C5K), as demonstrated by the fact that the C^α^ atoms superimpose with root-mean-square deviation (rmsd) of 0.54, indicating that TA_1–107_ binding to TolB does not result in any major conformational change in TolB; however, it should be borne in mind that the N-terminal residues 23–31 of TolB are not visible in the free TolB structure.

The molecular surface of the pocket in TolB in contact with residues 9–20 of TA_1–107_ is characterized by a mixture of hydrogen bonding donor and acceptors as well as hydrophobic patches (Tables S1 and S2). The intermolecular contacts are dominated by the interactions established by ColA residues that are essential for biological activity (Fig. 2). The first set of interactions are predominantly hydrophobic in nature, with only one hydrogen bond between W15 of ColA and D307 of TolB (Tables S1 and S2). Several non-polar contacts are also made by the W15 side-chain to residues P312, S306, T305, T291 of TolB, with additional interactions made by D11 and T13 with H245 and L268 of TolB respectively (Table S2). The second cluster of interactions consist of mainly polar contacts, with E18 making three direct interactions and one water-mediated contact to TolB, and S17 engaged in two further hydrogen bonds with TolB, one direct and one water-mediated that bridges the Ca^2+^ ion deep in the binding pocket (Fig. 3C; Table S3). It can be argued that residues W15 and E18 are interaction hot spots for the TA_1–107_–TolB interface and are likely to provide a marked contribution to the energy of association. Both side-chains of T13 and W15 become buried in the complex, excluding a significant area of TolB from the solvent and adding to the favourable energy provided by the total of 8 (5 direct) hydrogen bonding interactions that stabilize the association. All of the S17 and E18 interactions are buried into the binding pocket and appear to be virtually excluded from the bulk solvent, partly by the shielding from the side-chain of M203 of TolB, which makes non-polar contacts with the aliphatic portion of the E18 residue (Table S2). Complex formation with TA_1–107_ results in the burial of 531 Å^2^ of the TolB solvent accessible surface area which is less than the buried surface areas of TolB in complex with Pal and TE9_pep32–47_ (Table S4). Hydrophobic residues in the TolB pocket such as M203, F219 and L268, which are relatively solvent exposed in the free TolB molecule, become almost completely buried in the complex.

A total of eight direct (protein–protein) intramolecular hydrogen bonds exist in the structure of the TolB binding epitope of TA_1–107_ that involve both main chain and side-chain atoms (Table S5). For example, S16 engages in four intramolecular interactions, two via its side-chain and two via its main chain. One of these, with D11, is a strong contact with a distance of 2.6 Å and good geometry. This contact is likely to contribute to stabilizing the conformation of the bound TA_1–107_ as both residues have a profound effect on the interaction with TolB (Fig. 2). Nuclear magnetic resonance studies of the translocation domain of ColE9 have shown the presence of clusters of interacting residues of the TolB box that define a protein binding epitope which is subsequently weakened following the introduction of mutations to key residues within these clusters (Macdonald *et al*., 2004; Tozawa *et al*., 2005). Given the similarity of the core region of the TolB boxes of ColA and ColE9, and the complex and extensive network of intramolecular hydrogen bonds it is reasonable to assume that, despite the lack of a well-defined secondary structural element in this segment of ColA, part of the structure of this TolB binding motif might be pre-formed in solution. It appears, in fact, that the intramolecular interactions could define and stabilize, even though temporarily, a productive conformation in terms of TolB binding.

### Comparison with the interactions in the ColE9–TolB complex

**Fig. 4:**
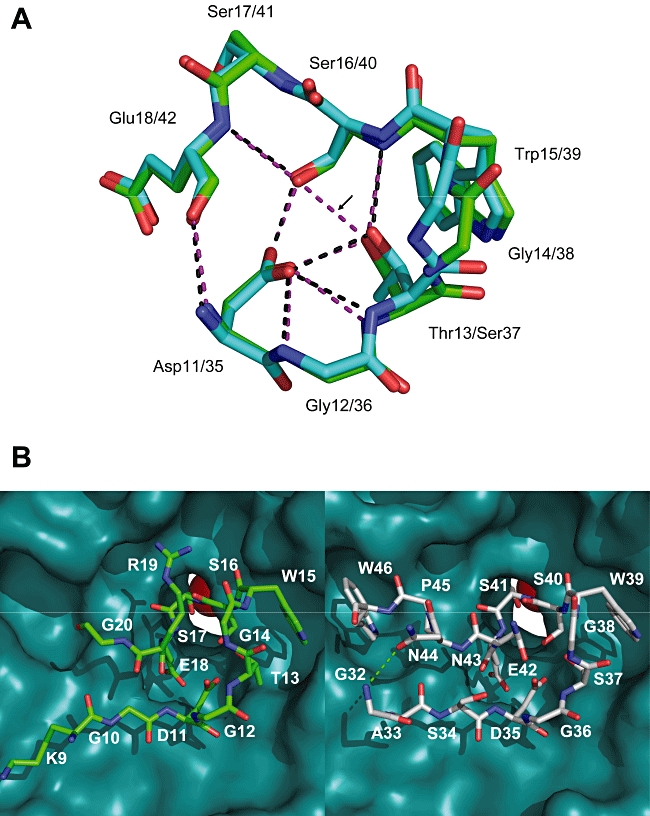
Comparison of TA_1–107_–TolB with TE9_pep32–47_–TolB (PDB entry 2IVZ). A. Ball and stick comparison of the stereochemistry of the core region of the TolB box of ColA (blue) with ColE9 (green). Intramolecular hydrogen bonds are shown in magenta dashed lines for ColE9 and black dashed lines for ColA. The hydrogen bond between S37 and S40 of TE9_pep32–47_–TolB (shown by the arrow) has no equivalence in TA_1–107_–TolB due to the small perturbation in the position of the T13 residue. B. Ball and stick representation of residues 9–20 of TA_1–107_ bound to the central canyon of the TolB β-propeller (left) in comparison with the TE9_pep32–47_–TolB interaction (right). The cyclizing hydrogen bond between G32 and N44 in ColE9 is shown as a green dashed line.

The structure of the complex of TolB and the ColE9-derived peptide TE9_pep32–47_ (2IVZ) demonstrated that the TolB box in ColE9 consists of a 15-residue peptide from 32-GASDGSGWSSENNPW-46 (Loftus *et al*., 2006). Our structure of the complex of TolB and TA_1–107_ has revealed that the TolB box in ColA is a 12-residue peptide with the sequence of 9-KGDGTGWSSERG-20. The TolB boxes in ColA and ColE9 share a core sequence of DG(S/T)GWSSE, which superimpose well in the two structures except the G(S/T)G residues which are separated slightly away in the TA_1–107_–TolB structure (Fig. 4A). This results in the intramolecular hydrogen bond between S37 and S40 in ColE9 being absent in the TA_1–107_–TolB structure.

The TolB box core of ColA forms an O ring shape, due to the hydrogen bond between the D11 and the E18 residues, and sits at the bottom of the tunnel in TolB (Fig. 4B) with six hydrogen bonds stabilizing the interaction with TolB (Table S1). The TolB box core region in ColA provides (i) all the intermolecular hydrogen bonds with TolB; (ii) seven out of eight intramolecular hydrogen bonds and all the hydrophobic interactions with TolB, except for one between K9 from ColA and F219 of TolB (Tables S2 and S5); and (iii) three out of four water-mediated interactions with TolB (Table S3). The additional C-terminal TolB box residues in ColE9, that are not found in ColA, provide an intermolecular hydrogen bond between W46 from ColE9 and L202 of TolB (Table S1); four intramolecular hydrogen bonds between G32 and N44, G36 and S34, G38 and N43, and S37 and S40 (Fig. 4B; Table S5); and hydrophobic interactions between the residues of A33, P45, W46 from ColE9 and P201, M203, F423, Q172, V170 and K422 of TolB respectively (Table S2). These additional contacts presumably explain why ColE9 binds TolB with higher affinity than ColA.

Two calcium ions are required to make the β-propeller tunnel of TolB electrostatically positive to facilitate binding of the TolB box of ColE9, which has a pI value of 3.7 at neutral pH (Loftus *et al*., 2006). There is only one calcium ion seen in the β-propeller tunnel in the structure of the TA_1–107_–TolB complex, and one sodium ion is far away from the tunnel between the α/β and β-propeller domains (Fig. 3B). Two basic residues K9 and R19 in the TolB box of ColA do not form any interactions with TolB but raise its pI value to 6.1, which could explain why only one calcium ion is required in the β-propeller tunnel of TolB to facilitate binding of TA_1–107_.

### ColA does not competitively recruit TolB

**Fig. 5:**
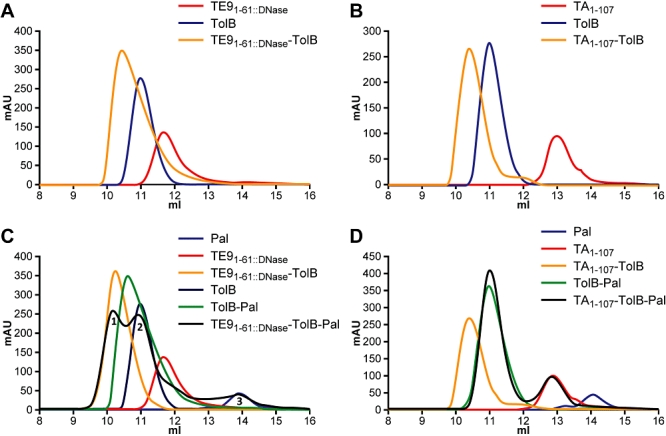
ColA binds to TolB but does not competitively recruit TolB from a TolB–Pal complex. A. Analytical gel filtration showing the individual protein peaks attributed to TolB, TE9_1-61::DNase_ and TE9_1-61::DNase_–TolB complex. B. Analytical gel filtration showing the individual protein peaks attributed to TolB, TA_1–107_ and TA_1–107_–TolB complex. C. Analytical gel filtration showing the protein peaks attributed to Pal, TE9_1-61::DNase_, the TolB–Pal and TE9_1-61::DNase_–TolB complexes, and the peaks produced from a mixture of TolB–Pal incubated stoichiometrically with TE9_1-61::DNase_ that shows the displacement of Pal (peak 3) as TolB is competitively recruited by TE9_1-61::DNase_ (peak 1). Residual TolB–Pal (peak 2) remains due to incomplete recruitment of TolB *in vitro*. D. In contrast when TolB and Pal were mixed together, incubated stoichiometrically with TA_1–107_, and run on gel filtration the absence of a protein peak with the same retention time as TA_1–107_–TolB demonstrates no competitive recruitment of TolB by TA_1–107_. Protein peaks attributed to Pal, TA_1–107,_ TolB–Pal and TA_1–107_–TolB are shown.

It has recently been shown that the binding surfaces of ColE9 and Pal with TolB are identical and that, in the presence of Ca^2+^, the TolB box of ColE9 competitively recruits TolB from Pal, presumably as a means of destabilizing the outer membrane on route to cell killing (Loftus *et al*., 2006). The *K*_d_ value of 1.6 µM for binding of TA_1–107_ to TolB in the presence of Ca^2+^ is much higher than the value of 90 nM reported for the binding of Pal to TolB in the presence of Ca^2+^ (Loftus *et al*., 2006), and thus makes it unlikely that the TolB box of ColA will competitively recruit TolB from the TolB–Pal complex. We confirmed this prediction by using analytical gel filtration chromatography. An interaction was observed between TolB and TE9_1-61::DNase_ (Fig. 5A), which is a chimeric polypeptide consisting of the first 61 residues of ColE9 fused to the DNase domain (Macdonald *et al*., 2004), or TA_1–107_ (Fig. 5B). However, when the pre-formed TolB–Pal complex was incubated, in the presence of Ca^2+^, with an equivalent concentration of TE9_1-61::DNase_ (Fig. 5C), or TA_1–107_ (Fig. 5D), only the ColE9 NDR was able to competitively recruit TolB as shown by a retention peak (peak 1) that overlapped the retention peak produced by TE9_1-61::DNase_–TolB, and a retention peak (peak 3) of free Pal (Fig. 5C). As TE9_1-61::DNase_–TolB and TolB–Pal have affinities of binding of 84 nM and 90 nM respectively, in the presence of 1 mM Ca^2+^ (Bonsor *et al*., 2007), recruitment of TolB *in vitro* is incomplete resulting in the presence of residual TolB–Pal (peak 2) when the TolB–Pal heterodimer is mixed with TE9_1-61::DNase_ stoichiometrically.

The difference in the ability of ColE9 and ColA to competitively recruit TolB is presumably the result of the seven differences in the amino-acid sequence of their TolB boxes (Fig. 1; Fig. S4). It has been observed that the W46 residue that is unique to ColE9 becomes buried in a pocket of the TolB surface and blocks access of Pal to its binding site on TolB (Bonsor *et al*., 2007). ColA does not possess this distal tryptophan residue in its TolB box (Fig. 1) and there is no occupancy of this surface pocket of TolB by any residue of the TolB box of ColA (Fig. 4B and Fig. S4). To test the importance of the distal tryptophan and other residues of the ColE9 TolB box in the competitive recruitment of TolB, we engineered TA_1–107_ by introducing seven mutations K9A, G10S, T13S, R19N, G20N, S21P and G22W to create a mutant of TA_1–107_ (YZ67) with a TolB box that is identical in sequence to that of ColE9 (Table 2). SPR experiments showed that the *K*_d_ of the interaction of YZ67 with TolB was 1.1 µM in the presence of EDTA and 92.1 nM in the presence of Ca^2+^ (Table 2), which are almost identical to the *K*_d_ values reported for TE9_pep32–47_ binding to TolB (Loftus *et al*., 2006). Corrected sensorgrams and residual plots for the YZ67–TolB interaction are shown in Fig. S1C. We further dissected the role of the seven mutations introduced in YZ67 by engineering TA_1–107_ to produce a series of mutant proteins in which a smaller number of the ColA TolB box residues were replaced with the equivalent residues found in the ColE9 TolB box (Table 2). The affinity of binding of the mutant proteins to TolB correlated well with the number of ColE9 residues introduced.

**Table 2:** Mutagenesis of the TolB box of TA_1–107_.

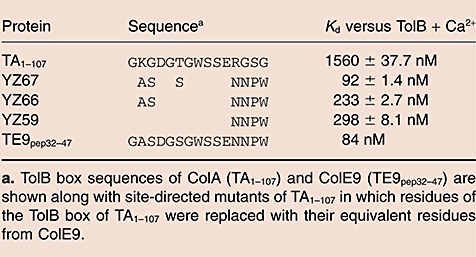

**Fig. 6:**
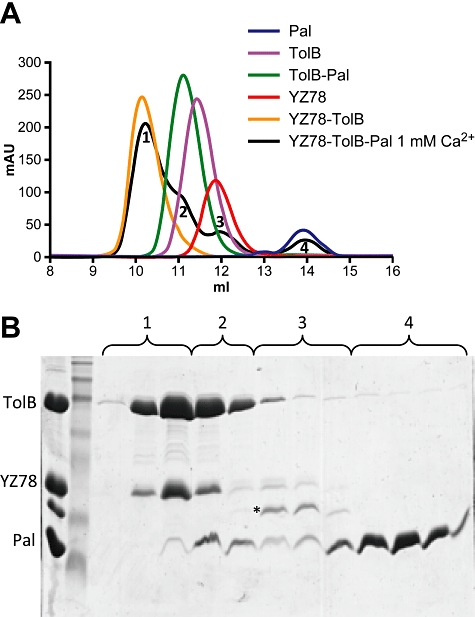
Competitive recruitment of TolB by ColA containing the TolB box of ColE9 (YZ78). A. Gel filtration of a mixture of TolB and Pal incubated stoichiometrically with YZ78 in the presence of 1 mM Ca^2+^. Peak 1 of the YZ78–TolB–Pal mixture superimposes with the YZ78–TolB control. B. Fractions collected across peaks 1–4 of the YZ78–TolB–Pal retention profile in A were analysed by SDS-PAGE. Peak 1 contained TolB and YZ78 indicating good separation of YZ78–TolB from any TolB–Pal. Peak 2 is a shoulder peak and contained a mixture of YZ78–TolB and TolB–Pal. Peak 3 contained uncomplexed YZ78, and peak 4 contained free Pal that had been displaced from the TolB–Pal interaction. *Indicates some breakdown product of YZ78 in the uncomplexed fractions.

To determine whether the increased affinity of the YZ67 mutant protein for TolB enables competitive recruitment of TolB from Pal, we used analytical gel filtration chromatography to observe the interaction of YZ67 with TolB in the presence of a stoichiometrically equivalent concentration of Pal, with or without Ca^2+^. In the absence of Ca^2+^ the high-molecular-weight retention peak (peak 1a) overlaps the peak produced by the TolB–Pal interaction (Fig. S5A), whereas with Ca^2+^ the high-molecular-weight peak (peak 1) shifts to the left superimposing with the peak produced by the interaction of YZ67–TolB (Fig. S5A). Analysis of these gel filtration traces by SDS-PAGE shows that in the absence of Ca^2+^, the proteins present in peak 1a are TolB and Pal, and in peak 2a are primarily YZ67 (Fig. S5B). In the presence of Ca^2+^, the major proteins present in the fractions of peak 1 are TolB and YZ67 but do contain some Pal, while peak 2 contains mainly low concentrations of all three proteins, and peak 3 contains Pal (Fig. S5B). There is a clear difference in the pattern of proteins in each gel filtration chromatogram that indicates competitive recruitment of TolB by YZ67 only in the presence of Ca^2+^. However, we were aware that the presence of Pal in the SDS-PAGE profile of peak 1 of Fig. S5A is consistent with either a trimeric YZ67–TolB–Pal complex or two separate heterodimers of TolB–Pal and YZ67–TolB running similarly and inseparably through the gel filtration column. To distinguish these two possibilities, we repeated the gel filtration experiments with TolB–Pal and YZ78 (a polypeptide that expresses the N-terminal 172 residues [TA_1-172_] of ColA with the TolB box of ColE9) to allow a greater separation of the YZ78–TolB and TolB–Pal dimers. The gel filtration profiles and SDS-PAGE of proteins in each gel filtration peak show the presence of a YZ78–TolB heterodimer, demonstrating the ability of YZ78 to competitively recruit TolB from Pal similar to the competitive recruitment seen by the TolB box peptide of ColE9 (Fig. 6).

**Fig. 7:**
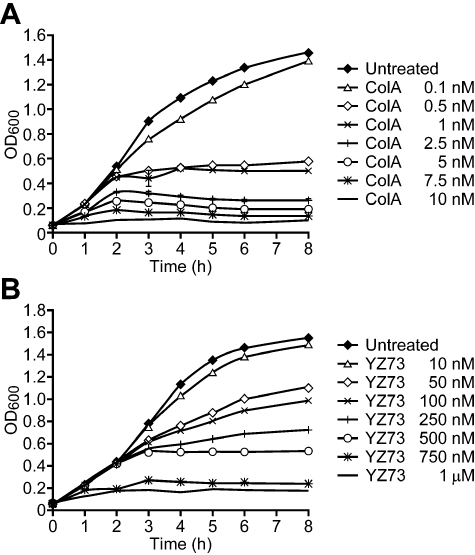
ColA expressing the TolB box of ColE9 (YZ73) has at least 100-fold less biological activity than ColA. Cell killing of *E. coli* DH5α in liquid culture following treatment with 0.1–10 nM of colicin A (A) and 10 nM to 1 µM of YZ73 (B). An untreated sample was included as a negative control for cell killing of both ColA and YZ73. Comparison of both panels suggests that 0.1 nM and 10 nM ColA had similar killing properties to 10 nM and 1 µM YZ73 respectively, while 1 nM of ColA had a killing activity similar to 500 nM of YZ73 indicating a reduced activity of YZ73 of, at least, 100-fold when compared with ColA.

Interestingly, activity assays of full size ColA containing the complete TolB box of ColE9 (YZ73) demonstrated that the biological activity of YZ73 was reduced by at least 100-fold when compared with ColA (Fig. 7). We therefore introduced the Y58A mutation, which has previously been shown to completely inactivate ColA (Pommier *et al*., 2005), into the TolA box of YZ73 and demonstrated that YZ73 containing the Y58A mutation was unable to interact with TolA by SPR, and was biologically inactive (data not shown). This confirmed the continued dependence on TolA for the translocation of YZ73 and the deleterious impact on biological activity of a TolB box with higher-affinity TolB binding. Even though YZ73 and ColA share the same number of residues between their respective TolA and TolB boxes, the differences in the nature of their interaction with TolB could result in changes in the relative geometry of their TolB and TolA binding epitopes.

## Discussion

Mutational and biophysical studies have indicated that translocation of ColA requires the same set of Tol proteins and outer membrane receptors as the enzymatic E colicins such as ColE9, even though (i) the cytotoxic domains of both types of colicin are different; (ii) translocation to different cellular locations is required for cell killing to occur; (iii) ColE9, unlike ColA, binds to sensitive *E. coli* cells as a complex with its immunity protein Im9; and (iv) there is no evidence that ColE9, unlike ColA, make any direct interaction with TolA. From the data presented in this paper we propose that Group A colicins use subtly different mechanisms to recruit the common TolB portal in order to gain entry into and then kill *E. coli* cells.

The cellular uptake of group A colicins involves the penetration of an NDR of their translocation domains through the cell envelope to make contact with the Tol proteins in the periplasmic space. In the case of ColE9 this ensures the competitive recruitment of TolB from the TolB–Pal complex by the binding of the TolB box of the colicin (Loftus *et al*., 2006; Bonsor *et al*., 2007). The structure of the TA_1–107_–TolB complex reported here shows that the TolB boxes of ColE9 and ColA interact in subtly different ways with the common TolB translocation portal (Fig. 4B), principally as a result of sequence differences in the distal half of the TolB box of each colicin. The shape complementary index values of 0.78 for the TE9_32-47_–TolB complex, 0.70 for the TolB–Pal complex and 0.66 for the TA_1–107_–TolB complex are in good agreement with the relative binding affinities of TolB for these three ligands and are consistent with the ability of ColE9, but not ColA, to recruit TolB from its complex with Pal. The ability to competitively recruit TolB was introduced into ColA when the complete TolB box of ColE9 was introduced into the ColA polypeptide, indicating the importance of the second tryptophan residue and the cyclizing hydrogen bond between N44 and G32 in the ColE9 sequence for high-affinity TolB binding. The role of competitive recruitment of TolB to the translocation of colicins is, at present, not well established especially allowing for the fact that TolB has been found as a monomer in the periplasm (Levengood and Webster, 1989) and bound to other outer membrane receptors such as Lpp and OmpA (Rigal *et al*., 1997; Clavel *et al*., 1998). TolB has also been shown *in vitro* to dimerize (Walburger *et al*., 2002), and forms a weak interaction with TolA (Dubuisson *et al*., 2002; Walburger *et al*., 2002; Hands, 2004). Therefore, it is possible that the ColA interacts with TolB directly in the periplasm or from a lower-affinity complex at the outer membrane.

The lower-affinity interaction of the TolB box of ColA with TolB, as compared with that of ColE9, may be important for the subsequent interaction of ColA with TolA, which is a component of the Brownian ratcheting process that is proposed to drive the unidirectional translocation of group A colicins (Journet *et al*., 2001). Our data show that the C-terminus of the TolB box of ColA is solvent exposed having no intermolecular interactions with TolB, which would facilitate an interaction with TolA by residues encompassing Y58-Y90 of the TolA box of ColA (Pommier *et al*., 2005). A trimeric complex produced by TolA–TolB–TA_1–107_ was disrupted when the TolB box of ColA (in TA_1–107_) was substituted with the TolB box of ColE9 (data not shown), suggesting that the higher-affinity complex of TolB with mutated TA_1–107_ (YZ67), mediated by the introduction of the K9A, G10S, T13S, R19N, G20N, S21P and G22W mutations affected the subsequent interaction with TolA. Indeed we show the importance of TolA to the activity of YZ73 following the introduction of the TolA box mutation, Y58A, into YZ73 which rendered YZ73 completely inactive.

The role of TolA in the import of enzymatic E colicins is less clear. It has recently been shown that TolA along with TolB are important in harnessing energy from the proton motive force for the release of Im9 from the ColE9 DNase/Im9 complex bound to *E. coli* cells (Vankemmelbeke *et al*., 2009). We assume that energy is not similarly required for the translocation of ColA as there is no immunity protein bound to the colicin when it is exported from the producing *E. coli* cell (Geli and Lazdunski, 1992).

Making use of the data reported in this paper, we propose subtly different models for the translocation of ColA and ColE9. The NDR of ColE9 contains two separate OmpF binding sites (Housden *et al*., 2005) and crosses the outer membrane through the lumen of an OmpF monomer (Kurisu *et al*., 2003; Yamashita *et al*., 2008). The entry of the ColE9 NDR into the periplasm disrupts the TolB–Pal complex through competitive recruitment of TolB by the TolB box. This would then allow TolB to interact with TolA leading to the pmf-dependent loss of Im9 from the DNase/Im9 complex and entry of the DNase domain through a destabilized outer membrane. Delivery of the DNase domain across the inner membrane as an intact molecule, or as a proteolytically cleaved subdomain (de Zamaroczy *et al*., 2001; Shi *et al*., 2005) is electrostatically driven and potentially involves the AAA^+^ ATPase FtsH (Walker *et al*., 2007). The translocation domain is stalled at this point as no other interactions of physiological significance with TolA, TolR or TolQ occur for enzymatic colicins (Hands *et al*., 2005; Cascales *et al*., 2007), and it has not been shown that any residues of the translocation domain distal to the NDR are able to enter the cell envelope of *E. coli* cells (Zhang *et al*., 2008). Unlike ColE9, the NDR of ColA does not have an OmpF binding motif and does not enter the OmpF lumen (Bainbridge *et al*., 1998). We suggest that the NDR slides down the side of OmpF (Lazzaroni *et al*., 2002) to make contact with TolB thus providing an anchor for a higher-affinity interaction with TolA. The affinities of ColA for TolB and TolA, respectively, are progressively higher (Hands *et al*., 2005) providing support for the Brownian Ratcheting hypothesis (Journet *et al*., 2001). It has also been shown that once bound to TolA, the ColA NDR cannot be displaced by TolB even with an excess concentration of the latter (Pommier *et al*., 2005), but a trimeric complex between all three proteins has been detected *in vivo* by an antibody overlay experiment (Bouveret *et al*., 1998) and *in vitro* by gel filtration chromatography (L. Chan, unpublished). The role of TolR in the translocation process remains unclear as the affinity of ColA for TolR is very low (> 15 µM; Hands, 2004), which suggests that it has no physiological significance, even though a TolR binding site in ColA has been proposed (Journet *et al*., 1999; Bouveret *et al*., 2002).

Support for this model has come from studies on the translocation of colicin N which also slides down the outside of OmpF through an interaction with lipopolysaccharide (Baboolal *et al*., 2008), and subsequently interacts with TolA, but not TolB, in the periplasm. It is not known why ColA, unlike ColN, interacts with TolB, while both ColA and ColN possess TolA boxes in their NDRs (Raggett *et al*., 1998; Pommier *et al*., 2005), a feature that is not present in the NDR of enzymatic colicins. The surprising observation that the translocation domain expressing residues 1–172 of ColA denatures TolAIII on binding (Deprez *et al*., 2002) is also of significance as it may lead to the destabilization of the cell envelope following the loss of the TolA–Pal transmembrane linkage and result in entry of the pore forming domain (Cascales *et al*., 2000).

Our data have shown that the lower affinity of binding and the smaller buried surface area of the TolB box of ColA with TolB are important features in the translocation of ColA and presumably facilitate the subsequent interaction of the TolA box of ColA with TolA. By mutating the TolB box residues of ColA to equivalent residues of ColE9 we have shown that increasing the affinity of the TolB box for TolB has a detrimental effect on the activity of the colicin leading us to predict that Group A enzymatic and pore-forming colicins have diverging mechanisms of cellular penetration even though they use a common TolB portal.

## Experimental procedures

### Plasmids, bacterial strains, and media

*Escherichia coli* DH5α was used as the host strain for cloning and mutagenesis. *E. coli* BL21 (DE3) (Novagen) was used as the host strain for the expression vector pET21a (Novagen). All cultures were routinely grown in Luria–Bertani (LB) broth or on plates of LB agar, supplemented where required with ampicillin (100 µg ml^–1^). Plasmid pYZ27 contains *caa* cloned into pET21a via NdeI and XhoI. Plasmid pTA107 was derived from pYZ27 by engineering a stop codon after residue 107 of ColA. Plasmid pYZ59 was derived from pTA107 by substituting the residues R_19_GSG_22_ for N_43_NPW_46_ of ColE9; pYZ66 was derived from pYZ59 by substituting the residues K_9_G_10_ with residues A_33_S_34_ from ColE9; pYZ67 was derived from pYZ66 by engineering the site directed mutation, T13S; pYZ73 was derived from pYZ67 and contains *caa* with the ColE9 TolB box. Plasmid pRJ379 was used for the production of TolB (Carr *et al*., 2000), and pSL13 encodes the soluble periplasmic domain of Pal (residues 65–173) with a C-terminal His_6_-tag (Loftus *et al*., 2006).

### Protein expression and purification

Proteins were overexpressed in BL21 (DE3) cells and purified using nickel affinity chromatography followed by gel filtration chromatography as described previously (Carr *et al*., 2000; Penfold *et al*., 2000). Protein nomenclature follows the plasmid designations, such that YZ67 was expressed from pYZ67 and YZ73 was expressed from pYZ73.

### Formation of the TA_1–107_–TolB complex

TA_1–107_ and TolB, both in 20 mM Tris.Cl pH 8.0 and 5 mM CaCl_2_, were mixed in equimolar amounts and left at 4°C overnight. The mixed sample was then loaded on a Superdex 75 column (Amersham Biosciences) coupled to an FPLC system (ΔKTA) pre-equilibrated with 20 mM Tris.Cl pH 8.0, 5 mM CaCl_2_, and run at a flow rate of 0.5 ml min^−1^. The elution fractions were checked by running 15% SDS-PAGE gels.

### Crystallization

The concentration of the sample was checked prior to setting up extensive crystallization screening with the Pre-Crystallization Test kit (Hampton Research) to determine the most appropriate protein concentration for crystallization trials. Three different concentrations were tested: 5, 10 and 15 mg ml^−1^, and the best results were obtained at 5 mg ml^−1^, which was then used for crystallization. The Hydra II microdispensing system (Robbins Hydra, Matrix Technologies Ltd, Wilmslow, England) was used in the initial high-throughput crystallization and screening process. The sparse-matrix factorial search method (Jancarik and Kim, 1991) was used for the screening of initial crystallization conditions, eventually extending the trials to all the following 12 screens (Nextal Biotechnology/Qiagen): Classics, MbClassics, PEGs, Anions, Cations, AmSO_4_, pH clear, MPD, JCSG+, PACT, PACTpremier, Protein Complexes. The sitting-drop vapour-diffusion method was employed and 96-well crystallization plates (Grenier, Molecular Dimensions) were used, equal volumes of protein and reservoir solution (1 µl each) were dispensed and combined into sitting drops. The plates were sealed tightly with clear tape and kept in an incubator at 20°C. Optimized crystals of the TA_1–107_–TolB complex were obtained at 20°C with 2 µl of the complex at 5 mg ml^−1^ in 20 mM Tris-HCl pH 8.0, 5 mM CaCl_2_ mixed in equal volumes with reservoir solution containing 20% PEG 10000, 0.1 M Hepes pH 7.5. The crystals grew after 3–4 weeks. Before flash-freezing in liquid nitrogen, crystals were briefly soaked in 22% PEG 10000, 0.1 M Hepes pH 7.5 and 20% glycerol for cryoprotection.

### Data collection and phasing

Diffraction data were collected at −180°C on a Rigaku R-AXIS IV^++^ detector using CuKα radiation from a Rigaku Micromax-007 rotating anode, equipped with Osmic VariMax HF optics and an X-stream 2000 cryocooling vapour jet. Data were indexed, integrated and scaled with the programs MOSFLM (Leslie, 1992) and SCALA (Evans, 1997) of the CCP4 (1994) suite. Crystals of the TA_1–107_–TolB complex belong to the monoclinic space group P1211 and contained one molecule in the asymmetric unit with a solvent content of 27.7%. Phases were determined by the molecular replacement method using the program Phaser (McCoy *et al*., 2005) in the CCP4 suite and the *E. coli* TolB structure with PDB entry 1C5K (Carr *et al*., 2000) was used as a search model, after all waters were removed from this file and B factors reset to 20 Å^2^.

### Model building and refinement

The model was built manually using COOT (Emsley and Cowtan, 2004) and then refined against the X-ray data to 2.6 Å resolution spacing using restrained and TLS refinement in REFMAC5 (Murshudov *et al*., 1997). The optimal number of TLS groups, 7, was determined using the TLSMD server (Painter and Merritt, 2006). The final model consists of TolB, with resolved residues from 33 to 431, and TA_1–107_, with resolved residues from 9 to 20; it has excellent geometry and no Ramachandran outliers. Data processing and model refinement statistics are summarized in Table 1. Surface area accessibility calculations were carried out using the program AREAIMOL (Lee and Richards, 1971). All structural figures were prepared using PYMOL (Delano Scientific). Attempts to collect higher-resolution synchrotron data were not successful.

### Colicin activity assay

Liquid growth and spot test assays of biological activity of ColA and ColA mutants were conducted as described previously (Hands *et al*., 2005).

### Surface plasmon resonance

Surface plasmon resonance was conducted using a BIAcore X instrument from BIAcore AB (Uppsala, Sweden), operating BIAcore control software. TolB was immobilized to the matrix of a newly docked CM5 sensor chip, pre-equilibrated in HBS-EP [10 mM Hepes (pH 7.4), 150 mM NaCl, 3 mM EDTA, 0.005% (v/v) P20 surfactant] running buffer (BIAcore AB), via amine coupling. To determine the R_max_ values of each analyte binding to the ligand, 50 nM TolB was injected across flow cell 2 of a CM5 chip previously activated with a mixture of 0.1 M ethyl-N-(3-diethylaminopropyl)carbodiimide (EDC, BIAcore AB) and 0.4 M N-hydroxysuccinimide (NHS, BIAcore AB) at a flow rate of 10 µl min^−1^ and a contact time of 3 min to produce an immobilization of 2100 response units (RU). Two minute injections of up to 20 µM ColA and TolB box mutants of ColA were then performed across the immobilized TolB at a flow rate of 30 µl min^−1^. After each analyte injection, the sensor chip surface was regenerated using a 2 min pulse of 10 mM glycine, pH 1.8. Each analyte/ligand interaction was repeated three times and the R_max_ values were determined in HBS-EP buffer and HBS-P, 1 mM CaCl_2_, using BIAevaluation software 3.1 to align all the binding curves.

To determine the binding affinity data, the R_max_ of SPR should not exceed 500 RU. Therefore, binding affinity data of each analyte to TolB was performed across a new TolB immobilized CM5 chip containing 420 RU of immobilized TolB using a range of analyte concentrations above and below the expected *K*_d_ of the interaction. Global analysis using BIA evaluation software 3.1 was used to fit corrected SPR responses to the theoretical 1:1 Langmuir binding model. Steady-state affinity data for corrected sensorgrams was also evaluated using the general fitting model of the BIAcore 3.1 software. The average equilibrium binding response (Req) for each sensorgram was calculated 50–100 s after injection, plotted against analyte concentration and fitted using the steady state affinity model (Biacore 3.1 software). The affinity data of TA_1–107_, YZ67 and TA_1–107_ inactive mutants were determined three times in HBS-P buffer containing 1 mM CaCl_2_.

### Analytical gel filtration

Protein samples were mixed and incubated overnight at 4°C. A Superdex 75 10/300 GL column was equilibrated with two column volumes (24 ml) of 50 mM Tris.Cl, pH 8 containing 0.15 M NaCl and 1 mM CaCl_2_ (or 3 mM EDTA) at a flow rate of 0.5 ml min^−1^. Proteins (30 µM in 100 µl) were loaded onto the column at a flow rate of 0.5 ml min^−1^ and collected as 500 µl fractions following changes in absorbance of the eluate at A_280nm_ with time. All fractions were analysed on SDS-PAGE.

### PDB deposition

Coordinates have been deposited in the Protein Data Bank (PDB ID Code, 3IAX).
