# Supplementary material for: The crystal structure of the TolB box of colicin A in complex with TolB reveals important differences in the recruitment of the common TolB translocation portal used by group A colicins
Source: Mol Microbiol. 2009 Aug 7;75(3):623–36. doi: 10.1111/j.1365-2958.2009.06808.x (PMC2821528; doi:10.1111/j.1365-2958.2009.06808.x)
Supplement: Supplementary file 1 [file mmi0075-0623-SD1.pdf]

Supplementary Material

for

The crystal structure of the TolB box of Colicin A in complex with TolB reveals important differences in the recruitment of the common TolB translocation portal used by group A colicins.

Ying Zhang, Chan Li, Mireille N. Vankemmelbeke, Philip Bardelang, Max Paoli, Christopher N. Penfold and Richard James

Supplementary Tables

Table S1. Inter-molecular polar contacts in the $TA_{1\text{-}107}$-TolB and $TE9_{pep32\text{-}47}$-TolB complexes.

Angle (°)	Distance (Å)	$TA_{1\text{-}107}$	TolB	TE9	Distance (Å)	Angle (°)
126	2.6	Glu 18 $O^{\varepsilon 2}$	Ser 204 O^{γ}	Glu 42 $O^{\varepsilon 2}$	2.5	124
131	3.0	Glu 18 $O^{\varepsilon 1}$	His 245 $N^{\varepsilon 2}$	Glu 42 $O^{\varepsilon 1}$	2.7	140
114	3.1	Glu 18 $O^{\varepsilon 2}$	Ala 248 N	Glu 42 $O^{\varepsilon 2}$	2.8	109
134	2.9	Ser 17 O^{γ}	Glu 292 $O^{\varepsilon 2}$	Ser 41 O^{γ}	2.8	130
134	3.4	Ser 17 N	Glu 292 $O^{\varepsilon 2}$	Ser 41 N	2.8	147
119	2.8	Trp 15 $N^{\varepsilon 1}$	Asp 307 $O^{\delta 2}$	Trp 39 $N^{\varepsilon 1}$	3.0	109
-	-	-	Leu 202 O	Trp 46 $N^{\varepsilon 1}$	3.0	152

Table S2. Inter-molecular non-polar contacts in the $TA_{1\text{-}107}$-TolB and $TE9_{pep32\text{-}47}$-TolB complexes.

$TA_{1\text{-}107}$	TolB	TE9
Lys 9 C^{γ}	Phe 219 $C^{\delta 2}$	Ala 33 C^{β}
	Pro 201 C^{β}-$C\gamma$	Ala 33 C^{β}
Asp 11 C^{β}	His 245 $C^{\delta 2}$	Asp 35 C^{β}
Thr 13 $C^{\gamma 2}$	Leu 268 $C^{\delta 1}$	Ser 37 C^{β}
Trp 15 C^{γ}-$C^{\delta 2}$-$C^{\beta 3}$	Pro 312 C^{β}-C^{γ}	Trp 39 C^{γ}-$C^{\delta 2}$-$C^{\beta 3}$
Trp 15 $C^{\eta 2}$-$C^{\zeta 2}$	Ser 306 C^{α}	Trp 39 $C^{\eta 2}$-$C^{\zeta 2}$
Trp 15 $C^{\eta 2}$-$C^{\varepsilon 3}$	Thr 305 $C^{\gamma 2}$	Trp 39 $C^{\eta 2}$-$C^{\varepsilon 3}$
Trp 15 $C^{\varepsilon 3}$	Thr 291 $C^{\gamma 2}$	Trp 39 $C^{\varepsilon 3}$
Ser 16 C^{β}	Thr 291 $C^{\gamma 2}$	Ser 40 C^{β}
Glu 18 C^{γ}-C^{α}	Met 203 S^{δ}-C^{ε}	Glu 42 C^{γ}-C^{α}
	Met 203 C^{ε}	Pro 45 C^{γ}
	Phe 423 C^{ζ}	Pro 45 C^{γ}
	Gln 172 C^{β}	Trp 46 $C^{\zeta 3}$
	Val 170 $C^{\gamma 1}$	Trp 46 $C^{\eta 2}$-$C^{\zeta 2}$
	Lys 422 C^{δ}	Trp 46 $C^{\zeta 2}$
	Pro 201 C^{β}	Trp 46 $C^{\delta 1}$

Table S3. Water mediated interactions between TA_{1-107} and TolB.

Distance (Å)	Angle (°)	TolB	Water	ColA	Distance (Å)	Angle (°)
2.8	114	His245 $N^{\delta 1}$	HOH 37 O	Gly10 O	2.6	148
2.8	110	Thr291 $O^{\gamma 1}$	HOH 33 O	Glu18 $O^{\varepsilon 1}$	3.0	144
			HOH 33 O	Asp11 $O^{\delta 2}$	2.6	127
2.9	121	Ser204 O^{γ}	HOH 59 O	Ser17 O^{γ}	2.8	126

Table S4. The buried surface areas between TolB and three binding ligands.

	TolB-ColA	TolB-Pal	TolB-ColE9 peptide
TolB buried $(Å)^2$	531	1142	598
Partner buried $(Å)^2$	667 (ColA)	1255 (Pal)	824 (peptide)
Complex buried $(Å)^2$	1198	2397	1422

Table S5. Intra-molecular polar contacts for TA_{1-107} and $TE9_{pep32-47}$ in their complexes with TolB.

TA_{1-107}			ColE9 (chain E)		
	Distance (Å)	Angle (°)		Distance (Å)	Angle (°)
			Gly 32 N Asn 44 $O^{\delta 1}$	3.2	135
Asp11 N Glu 18 O	2.9	151	Asp 35 N Glu 42 O	2.9	138
Asp11 $O^{\delta 1}$ Thr 13 $O^{\gamma 1}$	2.8	120	Asp 35 $O^{\delta 1}$ Ser 37 O^{γ}	2.6	115
Asp11 $O^{\delta 2}$ Ser 16 O^{γ}	2.6	127	Asp 35 $O^{\delta 2}$ Ser 40 O^{γ}	2.5	110
Thr 13 N Asp11 $O^{\delta 1}$	3.1	141	Ser 37 N Asp 35 $O^{\delta 1}$	3.0	126
Ser 16 N Thr 13 $O^{\gamma 1}$	3.1	131	Ser 40 N Ser 37 O^{γ}	3.0	131
Ser 16 O^{γ} Glu 18 N	3.2	117	Ser 40 O^{γ} Glu 42 N	3.0	118
Gly 12 N Asp11 $O^{\delta 1}$	3.0	113	Gly36 N Asp35 $O^{\delta 1}$	3.1	110
Ser 16 O Arg 19 N^{ε}	3.2	171	Ser 40 O Asn 43 N	3.1	114
			Gly 36 N Ser 34 O^{γ}	3.0	128
			Ser 37 O^{γ} Ser 40 O^{γ}	3.1	131
			Gly 38 N Asn 43 $O^{\delta 1}$	2.8	106

Supplementary Figure S1

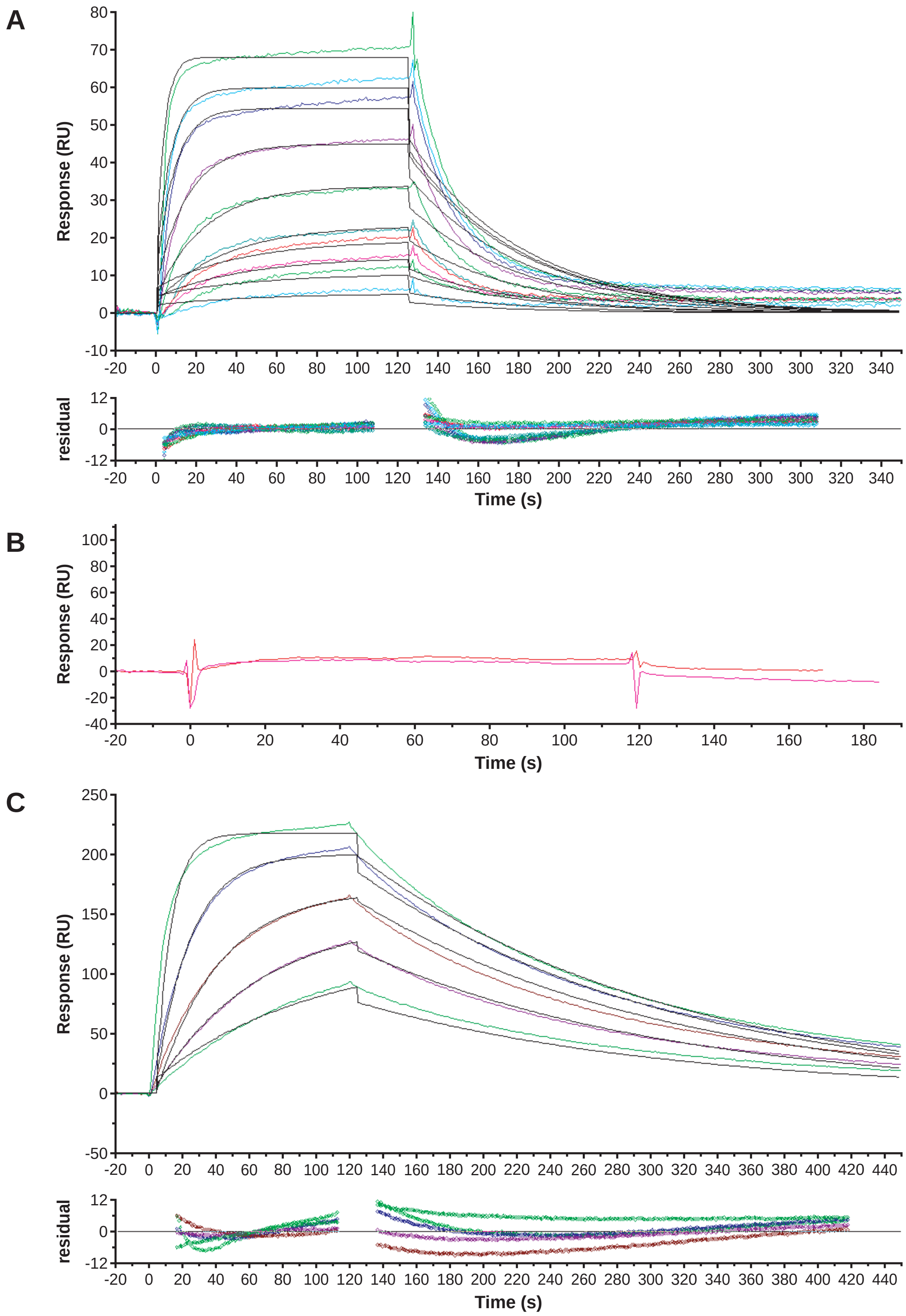

Fig. S1. TolB interaction with ColA determined by SPR. Corrected sensorgrams and residual plots of 0.1 µM, 0.2 µM, 0.4 µM, 0.5 µM, 1 µM, 2 µM, 5 µM, 8 µM, 10 µM, 20 µM $TA_{1\text{-}107}$ (A); 10 µM, 20 µM ColA E18A (B); 50 nM, 100 nM, 200 nM, 400 nM, 800 nM YZ67 (C) against TolB at 25°C with 1 mM Ca^{2+} are shown. The fitted kinetic data for binding are superimposed onto each sensorgram trace in panels A & C. The residual plots are shown underneath and highlight deviation of the experimental data from the theoretical fit. An interaction between TolB and ColA E18A shown in panel B cannot be detected, and no kinetic data or residual plots are therefore presented. The experimental data in panel A shows systematic deviation from the theoretical 1:1 Langmuir binding model throughout the dissociation phase. Therefore, in addition to global fitting, the equilibrium dissociation constant Kd for the interaction of $TA_{1\text{-}107}$ with TolB (A) was obtained using steady state affinity data (Biacore evaluation 3.1) which yielded a Kd value of 2.3 µM, similar to that reported in Table 2 using the global fitting.

Supplementary Figure S2

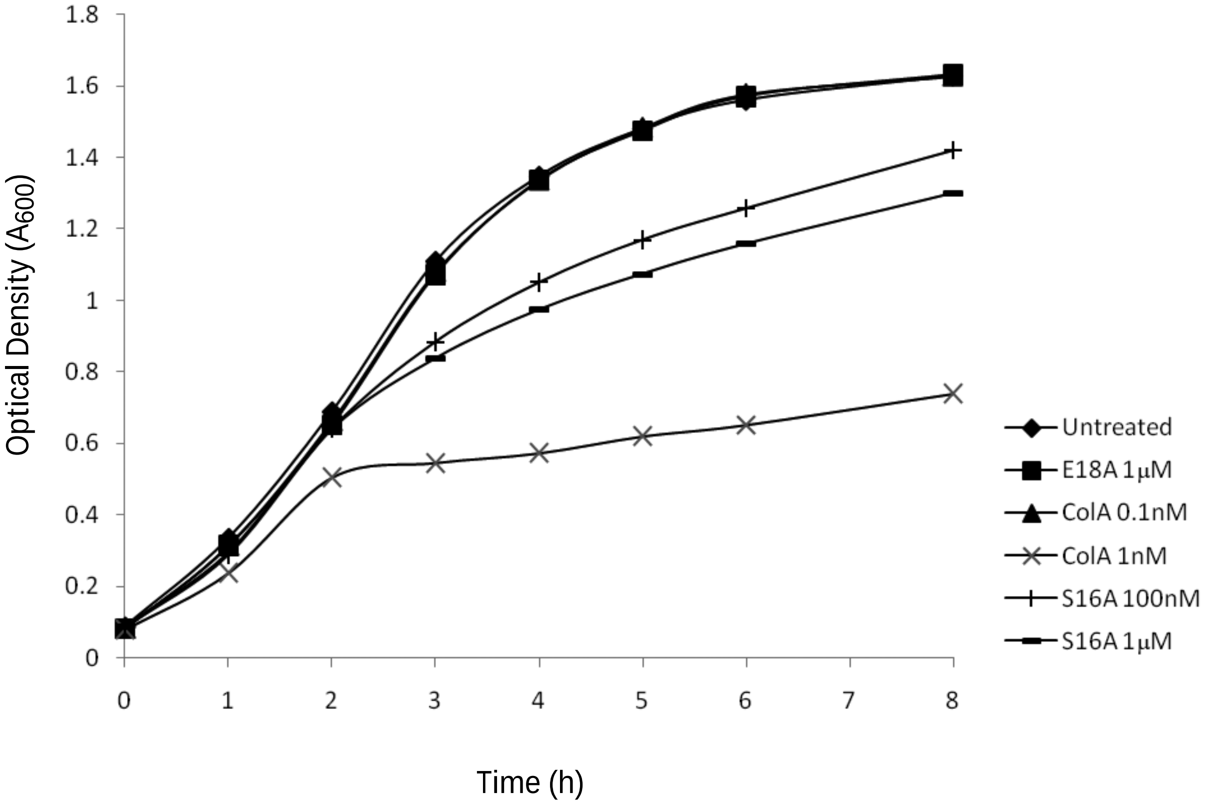

Fig. S2. ColA has >1000 fold greater biological activity than ColA S16A mutant. The amount of cell killing of *E. coli* DH5α in liquid culture following treatment with 1 µM of ColA S16A is less than 1 nM ColA. An untreated control and the inactive mutant ColA E18A were included as negative controls for cell killing. The data is a representative set of two independent experiments.

Supplementary Figure S3

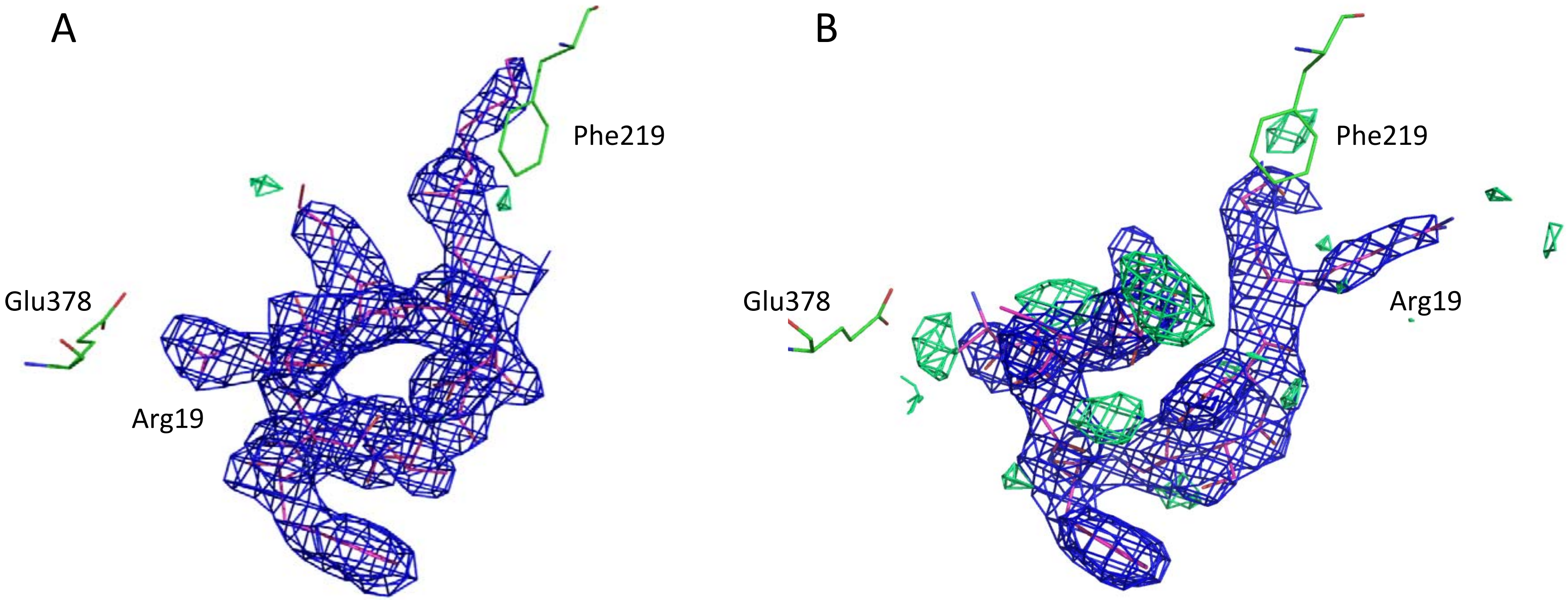

Fig. S3. Electron density map of the orientation of the TolB box of colicin A relative to TolB. 2Fo-Fc map contoured at 0.95σ is shown in blue; Fo-Fc map contoured at 3.0 σ is shown in green. Two residues Phe219 and Glu378 from TolB and one residue Arg19 from the TolB box are chosen as references. (A) shows the correct orientation of the TolB box with no unregistered extra density; (B) shows the incorrect orientation of the TolB box containing some unregistered extra density shown in green.

The TolB box of colicin A was built by fitting Trp15 into the big electron density pump of the extra density. The peptide was extended until the end of the extra density according to the sequence of the TolB box. Then the Refmac5 was run and the output map was checked in Coot that gave some extra electron density (B). We then changed the orientation of the peptide with Trp15 as the axis and the positive electron density matched with the residues of the TolB box, which gave no extra electron density (A).

Supplementary Figure S4

A

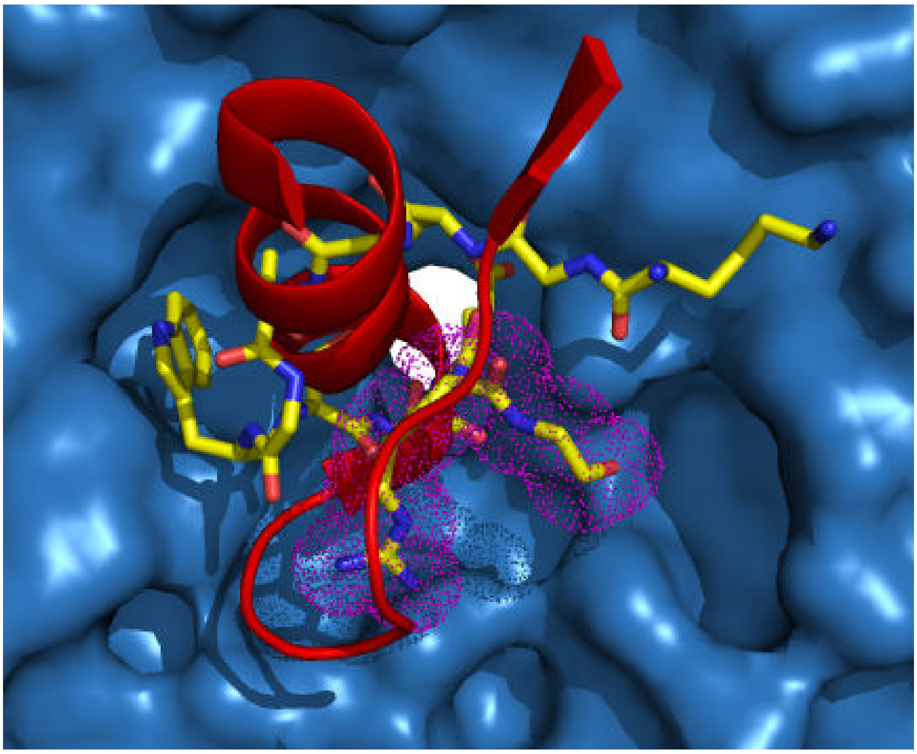

B

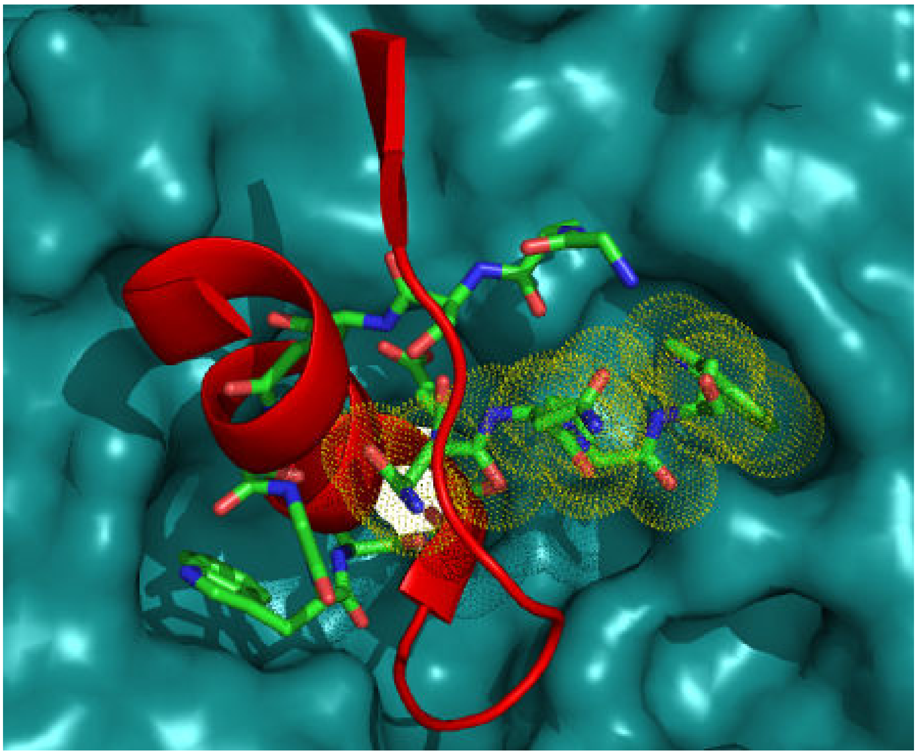

Fig. S4. Comparison of the TolB boxes of ColA and ColE9 in contact with TolB. Ball and stick representation of the TolB box residues of ColA (A) and ColE9 (B) in contact with TolB highlighting differences in the spatial distribution of distal residues of ColA (pink) and ColE9 (yellow) across the binding groove of TolB that influence competitive recruitment of TolB from Pal (red) by ColE9 but not ColA.

Supplementary Figure S5

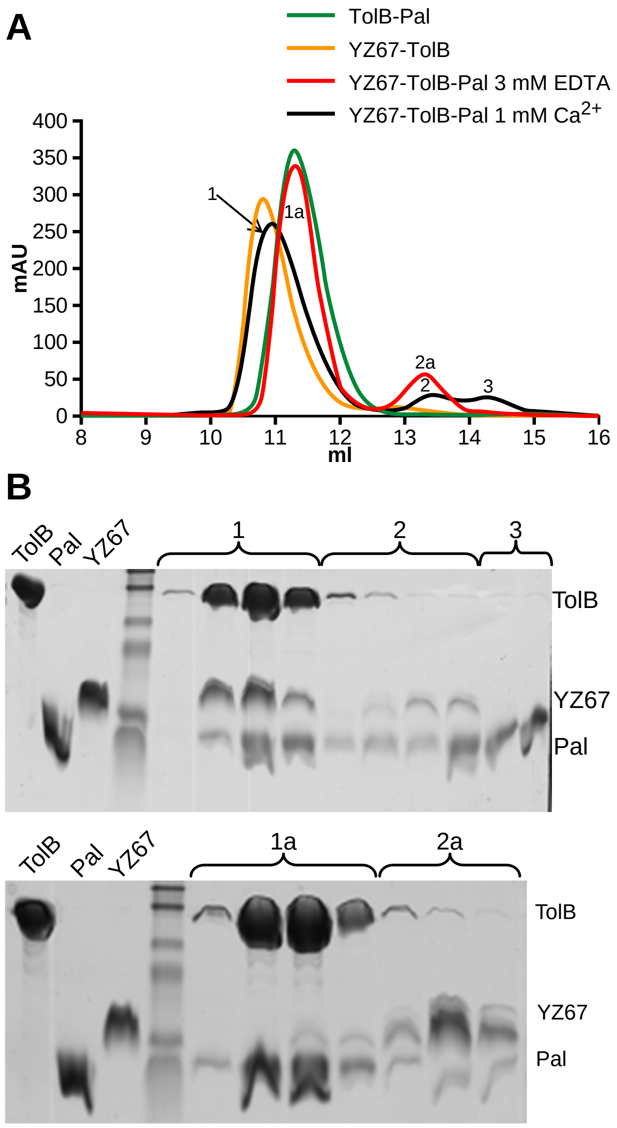

Fig. S5. ColA containing the complete TolB box of ColE9 competitively recruits TolB from Pal. (A) Gel filtration of a mixture of TolB and Pal, co-incubated stoichiometrically with YZ67 in the presence and absence of Ca^{2+} ions. In the presence of Ca^{2+} ions, three gel filtration peaks are observed (1-3) compared with only two in the absence of Ca^{2+} ions (1a, 2a). (B) Gel filtration fractions were collected across peaks 1-3 (top gel) and 1a-2a (bottom gel) and run on 16% SDS-PAGE. The appearance of YZ67 in peak 1 but not peak 1a indicates that YZ67 competitively recruits TolB from the TolB-Pal complex in the presence of Ca^{2+}.
